# Mechanism of Interaction of Water above the Methylammonium Lead Iodide Perovskite Nanocluster: Size Effect and Water-Induced Defective States

**DOI:** 10.1021/acs.jpclett.3c02807

**Published:** 2024-01-10

**Authors:** Jie Huang, Bowen Wang, Hejin Yan, Yongqing Cai

**Affiliations:** Joint Key Laboratory of Ministry of Education Institute of Applied Physics and Materials Engineering, University of Macau, Macau, China

## Abstract

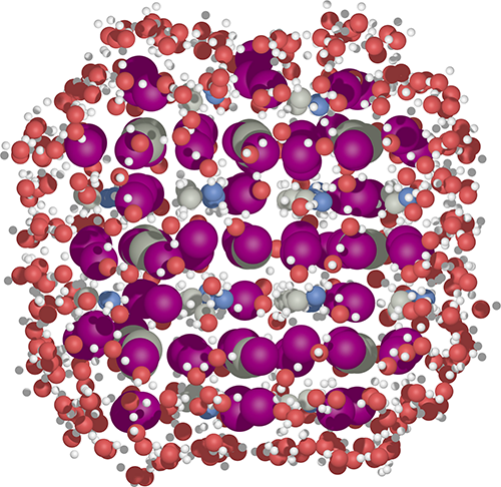

Water is often viewed as detrimental to organic halide perovskite stability. However, evidence highlights its efficacy as a solvent during organic perovskite liquid synthesis. This paradox prompts an investigation into water’s influence on perovskite nanoclusters. Employing first principle calculations and *ab initio* molecular dynamics simulations, surprisingly, we discover some subsurface layers of methylammonium lead iodide (MAPbI_3_) nanoclusters exhibit stronger relaxation than surface layers. Moreover, a strong quantum confinement effect enhances the band gap of MAPbI_3_ as the nanocluster size decreases. Notably, the water molecules above MAPbI_3_ nanoclusters induce rich localized defect states, generating low-lying shallow states above the valence band for the small amounts of surface water molecules and band-like deep states across the whole gap for large nanoclusters. This work provides insights into water’s role in the electronic structure and structural evolution of perovskite nanoclusters, aiding the design of water-resistant layers to protect perovskite quantum dots from ambient humidity.

Hybrid organic–inorganic perovskites (HOIPs) stand out as the most promising materials for next-generation solar cells due to their combination of low cost, high efficiency, easily tunable bandgap, extensive exciton diffusion length, and remarkable charge-carrier mobility.^1−8^ A HOIP follows the formula ABX_3_, where A represents an organic cation, B denotes a metal cation (i.e., Pb and Sn), and X signifies an anion (halogen). Among all the HOIP materials, methylammonium lead iodide MAPbI_3_ perovskite, where MA denotes CH_3_NH_3_^+^, has attracted significant attention because of its exceptional light-absorbing characteristics.

Unfortunately, HOIPs such as MAPbI_3_ suffer from the issue of instability which presents a significant challenge that constrains the applications.^4,5,8^ Exposure to environmental molecules, i.e., moisture and oxygen, induces the degradation of the structure. Under working conditions, the presence of heat and light irradiation would deteriorate the situation and accelerate the degradation.^9−12^ In particular, water, highly likely to be introduced during synthesis, storage, and serving processes, poses a significant effect on the stability and performance of perovskite materials.^13−16^ Subjecting MAPbI_3_ solar cells to relative humidity levels exceeding 55% rapidly impairs device performance.^17−21^ Consequently, maintaining the long-term stability of the MAPbI_3_ perovskite structure in moist conditions has become a central focus of research.^22−25^

Ironically, water can be intentionally introduced for growing HOIPs in the solution-based synthesis method of MAPbI_3_;^26,27^ thus a dual role is made by water. As the most clean and nontoxic solvent, water was found to be a promising solvent or cosolvent to make a homogeneous precursor solution.^28,29^ During the formation and crystallization of MAPbI_3_, proper control of moisture has been found to modulate the thin film morphology and improve the performance of solar cells.^13,30−37^ In contrast to other solvents commonly used for perovskite precursors, water does not fit neatly into the category of strongly coordinating solvents for lead ions due to its limited capacity to solvate PbI_2_ and its moderate donor number.^27^ Nevertheless, the high polarity of water molecules makes it challenging to produce iodide-rich iodoplumbates.^27^ How water would affect the structure and properties of MAPbI_3_ remains elusive, especially for those seeding MAPbI_3_ nanoclusters. The adsorbing behavior of water on perovskite nanoclusters remains an open question.

In this work, aiming at uncovering the effect of water, we perform density functional theory (DFT) calculations on MAPbI_3_ clusters. By tracking the variation of structures with *ab initio* molecular dynamics (AIMD) simulations, we provide nanoscale mechanisms of bond distortion and relaxation upon the uptake of water molecules. Moreover, we also examine the quantum size effect on band gaps of MAPbI_3_ nanoclusters and explore how water affects the electronic properties, i.e., identifying any defective states arising from water adsorption. Our work provides insight into the impact of water at the microscopic level on the structure of MAPbI_3_ clusters and holds immense potential for enhancing our understanding of degradation mechanisms and for future optimization of HOIPs.

Three different-sized nanoclusters, MA_7_Pb_8_I_36_, MA_32_Pb_27_I_108_, and MA_81_Pb_64_I_240_, with sizes of 1.50, 2.13, and 2.76 nm, respectively, were constructed by using Atomic Simulation Environment (ASE).^38,39^ For convenience, we refer to these nanoclusters as small, middle, and large, respectively. Initial atomic configurations for the three systems are shown in Figure 1a–c, with the nanoclusters built from the relaxed bulk MAPbI_3_ structure. For each case, a monolayer of water molecules was placed surrounding nanoclusters, with 156, 370, and 891 water molecules for the small, middle, and large systems, respectively. Water molecules are evenly distributed on the grids based on the density of bulk water at 300 K. The minimum distance between the oxygen atoms in water molecules and the atoms of MAPbI_3_ nanoclusters was set to 2.5 Å. A vacuum layer with a thickness of 5.5 Å was inserted to avoid the interaction between images of the nanoclusters under periodic boundary conditions. The optimized configurations for the three systems are shown in Figure 1d–f. The simulation settings and adsorption energies are detailed in Table 1.

**Figure 1:**
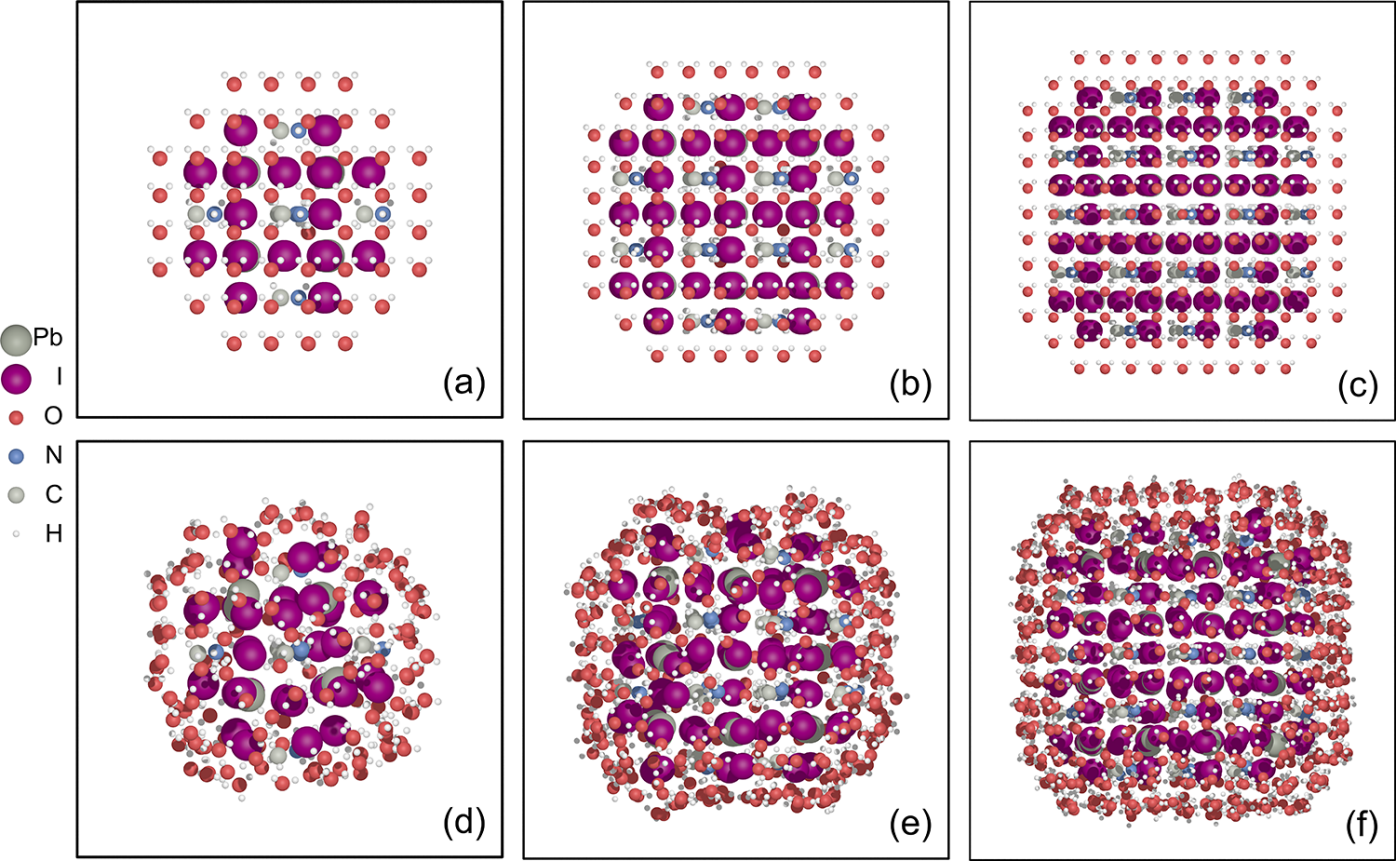
Initial (a, b, c) and corresponding relaxed (d, e, f) configurations of three different sized MAPbI_3_ nanoclusters surrounded by a layer of water molecules.

**Table 1:** Simulation Settings: the Cluster Sizes, Water Molecule Numbers, Adsorption Energies in Units Atomic Unit of Energy (a.u.), Highest Occupied Molecular Orbitals (HOMOs), Lowest Unoccupied Molecular Orbitals (LUMOs), and Band Gaps of the Naked MAPbI_3_ Nanoclusters^a^

	Small	Middle	Large
Cluster	MA_7_Pb_8_I_36_	MA_32_Pb_27_I_108_	MA_81_Pb_64_I_240_
Cluster size (nm)	1.50	2.13	2.76
H_2_O number	156	370	891
Atom number	568	1501	3625
Box volume (nm^3^)	30.04	49.13	90.74
*E*_adsorb_ per H_2_O (a.u.)	–0.0174	–0.0131	–0.0100
HOMO (eV)	0.593	0.113	0.582
LUMO (eV)	2.565	1.815	1.065
Band gap (eV)	1.972	1.702	0.484

a The cluster sizes were obtained by averaging the diameters in three directions. The adsorption energies were calculated by *E*_adsorb_ = *E*_total_ – *E*_cluster_ – *E*_water_, where *E*_total_, *E*_cluster_, and *E*_water_ are energies of the relaxed configuration of nanocluster and water, naked nanocluster, and water clusters, respectively. For further details regarding the adsorption energy calculations, please refer to Table S1 in the Support Information (SI).

DFT calculations and AIMD simulations were conducted using CP2K/QUICKSTEP,^40^ employing Goedecker–Teter–Hutter (GTH) pseudopotentials^41,42^ with single-zeta sets (SZV-MOLOPT-GTH for C, N, O, and H; SZV-MOLOPT-SR-GTH for Pb and I), the Perdew–Burke–Ernzerhof (PBE) functional,^43^ and DFT-D3 correction^44^ for the dispersion interaction. The Broyden–Fletcher–Goldfarb–Shanno (BFGS)^45−48^ optimizer is employed for small and midsized systems, while the Limited-memory BFGS (L-BFGS)^49^ optimizer is utilized for the large system. AIMD simulations were performed with canonical NVT ensembles with a Nosé–Hoover^50^ thermostat at 300 K for 12 ps using a time step of 1 fs. And MDAnalysis^51,52^ was used to analyze AIMD trajectories.

As shown in Figure 1, in comparison to the initial structures, optimized MAPbI_3_ clusters covered by water exhibit a certain degree of expansion. However, the extent of expansions is limited, essentially largely maintaining clusters’ octahedral structures. The surface water in the outer layer of the optimized structure forms a hydrogen bonding network that evenly envelops the clusters. (Figure S5 in SI shows the hydrogen bonding network.) We are particularly interested in the structural changes of MAPbI_3_ after the introduction of the water layer, in other words, geometrical modifications of bonds before and after relaxation. We thus monitor the distribution of the Pb–I distance.

In Figure 2, the three subplots (a), (b), and (c) show the distributions of Pb–I distance of the unrelaxed and relaxed structures for the three clusters. As can be seen from Figure 2, for the three cases directly cut from bulk MAPbI_3_ (the unrelaxed cases), the Pb–I bond distributes quite narrowly with peaks at around 3.15 Å, ranging from 3 to 3.25 Å. However, after relaxation, the peak broadens significantly and the values of Pb–I bond length extend from around 3.00 to 4.50 Å. This reflects a strong relaxation and distortion of the perovskite units upon forming nanoclusters. The presence of long Pb–I bonds greater than 4 Å indicates the presence of significant stress and strain inside these structures. We also find that the smaller the nanocluster the higher the population of those elongated Pb–I bonds. The broadened peaks of the Pb–I distance distributions suggest the presence of an elongated and stretched lattice surrounding the Pb atoms. We can identify discrete peaks in the extending distribution of Pb–I bonds, specifically within the Pb–I distance range of 3.0 to 3.5 Å, indicating the presence of the shell-like elongated lattice surrounding the center.

**Figure 2:**
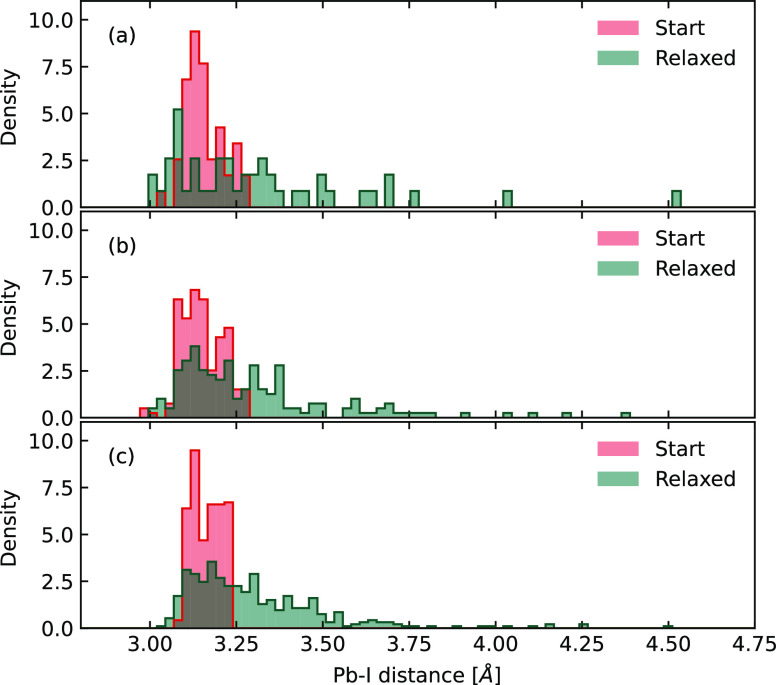
Pb–I distance distributions before and after relaxation for small (a), middle (b), and large (c) nanoclusters. The vertical axis indicates the probability density, which is defined as ρ_*i*_ = *n_i_*/(ND_*i*_), where *n*_*i*_ is the number of Pb–I distances in the *i*-th bin range, *N* is the total number of counted distances, and *D*_*i*_ is the width of the *i-*th bin. The area under the histogram integrates to 1, i.e., ∑ρ_*i*_*D*_*i*_ = 1.

To provide further spatial dependence of the relaxation from the surface to the center, we analyze the variation of the Pb–I distance for all of the Pb–I bonds before and after relaxation. As shown in Figure 3, the radial positions of all the I atoms, with their coordinates relative to the mass center of the cluster, are depicted together with the change of bond length, ΔPb–I_dis_. A positive (negative) value indicates that the bond is stretched (compressed). The probability densities for the distribution of the change in Pb–I distance are also plotted on the right side of these plots in Figure 3.

**Figure 3:**
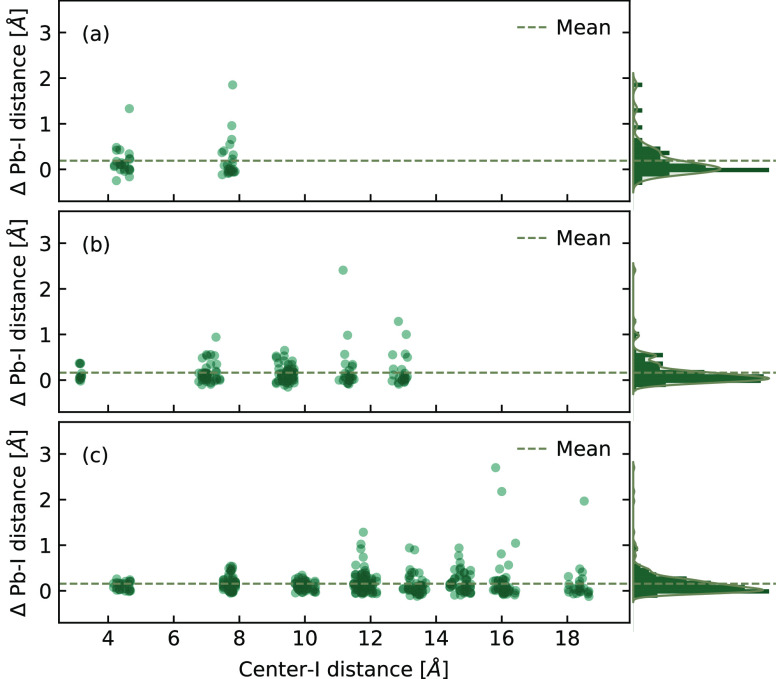
Spatially resolved distribution of the change in Pb–I distance for small (a), middle (b), and large (c) nanoclusters. The horizontal axis is the position of the I atom from the mass center of the corresponding nanocluster. The dashed lines correspond to the average values of the change in the Pb–I distance.

In all three sizes, the average values (dashed lines) of change in the Pb–I distance are positive, indicating that more than half of the Pb–I bonds in the nanocluster are stretched compared to the bulk phase. Squeezed Pb–I bonds are also detected in each shell defined by the iodine atoms. The coexistence of elongated and compressed Pb–I bonds shows a strong Jahn–Teller distortion^53^ of the octahedral structures. Interestingly, the most significant relaxation in middle and large systems does not occur in the outermost layer, as usually assumed in the bulk surface, which interacts directly with the water molecules. Instead, the strongest bond elongation is found in the subsurface layers, extending to four iodine subsurface layers. This finding suggests that the outermost layer serves as chemical passivation and is water resistant, while the subsurface layers, with the most bond relaxation, undergo severe bond stretching. The outermost passivation layer, subjected to lattice discontinuity, largely maintains the octahedral lattice of the nanoclusters while resisting the water, as illustrated in Figure S1, fourth column, in the SI. In contrast, the interior atoms close to the center tend to deviate slightly from their bulk positions. As the MAPbI_3_ size increases, the change in Pb–I distance of the innermost layers decreases from the range [−0.28, 1.34] to [−0.02, 0.28], which indicates that the inner structure of a larger MAPbI_3_ nanocluster is much harder to disturb compared to a small one. Therefore, presumably, the loose subsurface structures are the origins of structural instability. How to effectively stabilize them is the key to promoting the population of seeds and the growth of high-quality nanoclusters.

To better understand the effects of water molecules on MAPbI_3_ clusters, we explored the perovskite–water ratio impact on the structure of the MAPbI_3_ cluster. Starting from a system like Figure 1a, we systematically reduced the number of surrounding water molecules from 156 to 0. For further details regarding the effect of the perovskite–water ratio, please refer to Figure S1 and Table S2 in the SI. Through our calculations, we notice that the small nanocluster would lose its octahedron structure during the structural relaxation process if there are no surrounding water molecules, while the basic crystal structure was preserved for the same nanocluster but with a small amount of water molecules. (Please refer to Figure S2 in the SI for the structure optimization processes of both systems.) Hence, water molecules surrounding nanoclusters play a certain role in stabilizing the Pb–I lattice and promoting the probability of seeding of the nanoclusters.

We next examined the size-dependent electronic property of MAPbI_3_ nanoclusters and the role of the water layer adsorbed around the surface. We attempt to answer the following questions. First, how does the band gap of MAPbI_3_ nanoclusters evolve with the size? Second, what is the effect of the surface water layer on the band gap? Are there any water-induced defect states? The projected density of states (PDOS) of the intrinsic nanoclusters, i.e., the structure without the surrounding water molecules, is shown in Figure 4. As the size of the nanocluster increases from 1.50 to 2.76 nm, the band gap decreases from 1.97 to 0.48 eV, consistent with the quantum confinement effect. The spatial confinement of the electrons and holes in small nanoclusters results in an increase in the energy spacing between the valence and conduction bands. The details of the highest occupied molecular orbital (HOMO) and the lowest unoccupied molecular orbital (LUMO) are given in Table 1. Notably, there exists a strong drop of the band gap from 1.70 eV for MA_32_Pb_27_I_108_ to 0.48 eV for MA_81_Pb_64_I_240_. This would arise from an improved stabilization of the octohedral unit at the inner core, as shown in Figure 2, with the atomic arrangement being more similar to the bulk lattice. It seems that the size of MA_81_Pb_64_I_240_ would be a critical value for becoming a bulk-like nanocluster. In addition, for all the intrinsic nanoclusters, there is no defective state in the gap regardless of their high surface ratio, rich surface dangling atoms, and strong distortion of bonds, indicating a good defect tolerance of MAPbI_3_.

**Figure 4:**
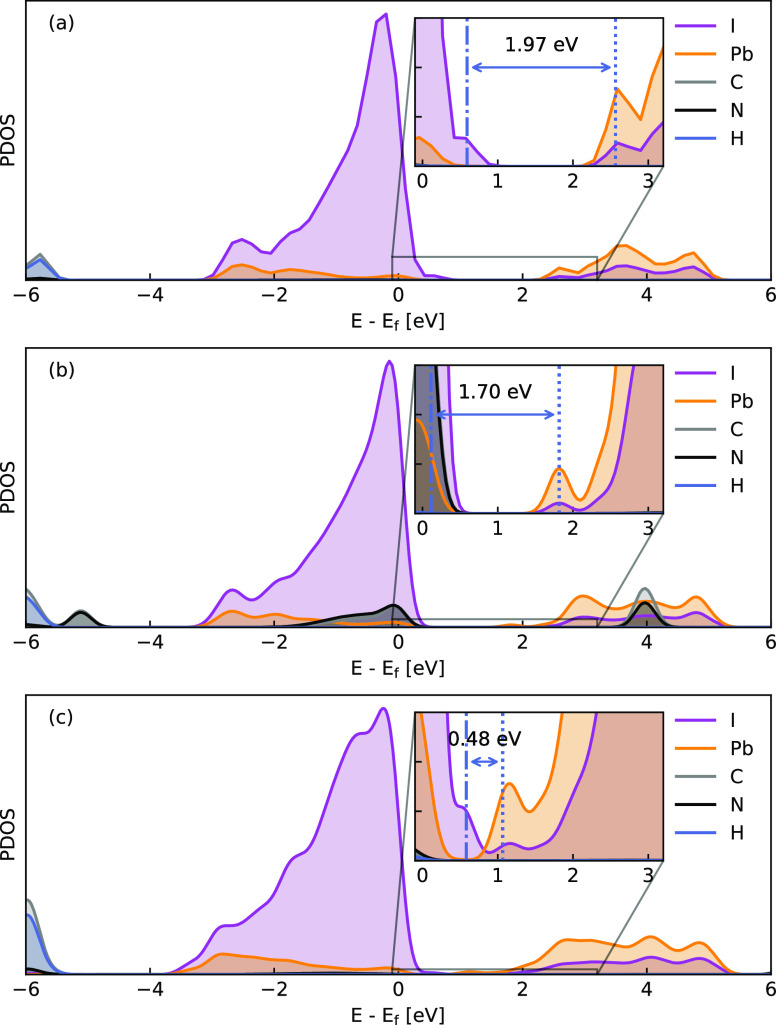
PDOS of the relaxed MAPbI_3_ clusters without adsorbing water molecules on the surface with small (a), middle (b), and large (c) sizes. The band gaps are 1.97, 1.70, and 0.48 eV, respectively.

With the addition of water molecules above the cluster surface, defective states formed within the band gap of MAPbI_3_. The introduction of water molecules results in the emergence of defect states in the MAPbI_3_ nanoclusters. By comparing the PDOS plots of nanoclusters without water molecules (Figure 4) and with water molecules (Figure 5), we find even for the smallest perovskite there are water-related states lying above the valence band top of MAPbI_3_. PDOS analysis shows that those states are mainly comprised of water and iodine. The strong overlapping of both states suggests a clear resonance of the water and surface iodine species and an overlapping of water and iodine orbitals.

**Figure 5:**
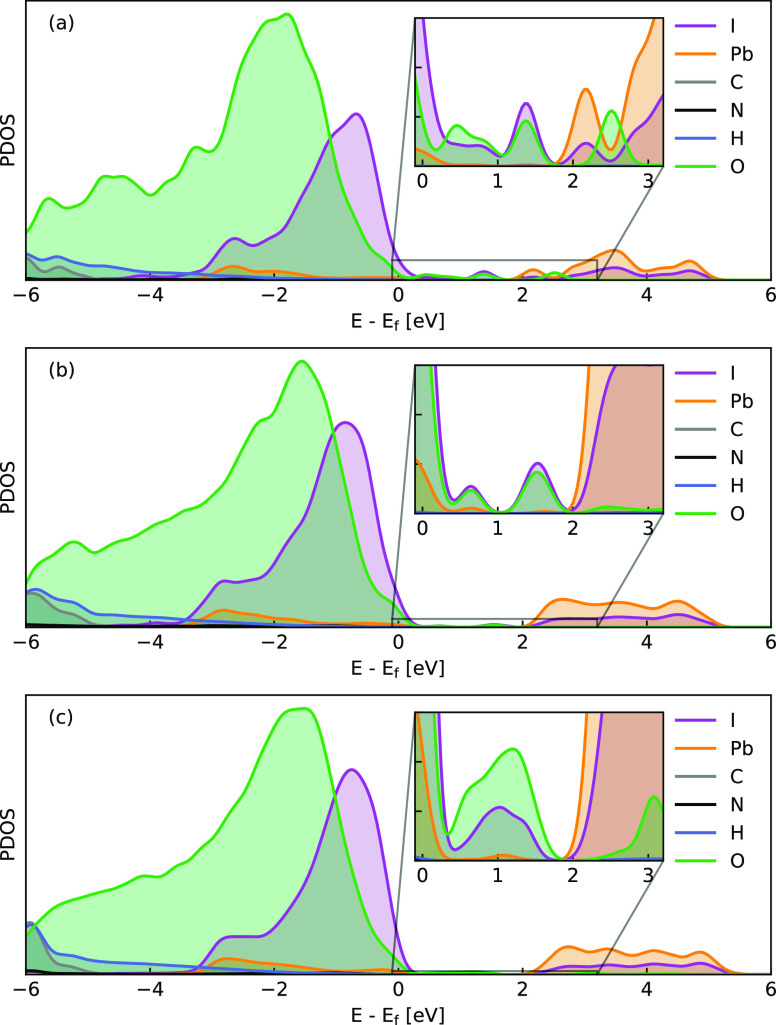
PDOS plots for the relaxed MAPbI_3_ clusters including water molecules in small (a), middle (b), and large (c) sizes. Defect densities of states can be found in all systems. We can observe that the most significant contributions to the defect density of states come from the atoms of the O and I atoms.

The effect becomes particularly pronounced when a significant number of water molecules come into contact with the nanoclusters, which occurs with a higher concentration of water molecules. Notably, the width of these water-induced states increases with the size of the nanocluster or equivalently with the surface area of the water layer. The low-lying states formed in the small nanocluster, narrow and shallow above the valence band, now broaden into band-like states across the whole band gap in the big nanocluster. The size-dependent behavior of the defective state suggests its origin from H_2_O molecule rather than the distortion of octohedra of Pb–I backbone. The low-lying shallow state above the valence band further indicates that each adsorbed H_2_O molecule acts as a p-type dopant, taking electrons from MAPbI_3_. In ultraviolet photoelectron spectroscopy (UPS) experiments, it was reported that the spectrum of MAPbI_3_ shows a blue shift after the sample is exposed to water.^54^ The experimental findings align well with the situation in which I atoms bind with water and lose electrons. This result suggests that water plays a crucial role in the electronic properties of MAPbI_3_ clusters by affecting their density of states and carriers concentration. It is noteworthy that the presence and relative position of such defective levels are less likely to be changed with the inclusion of spin–orbital coupling in the calculation, which, however, is not included here.

Therefore, besides the well-known degradation, water molecules above MAPbI_3_ nanoclusters can cause severe outcomes, including a negative impact on the efficiency of solar cells. A narrower band gap reduces the amount of energy that can be harvested from the solar spectrum, resulting in a lower energy conversion efficiency for the solar cell. These water-induced defective states, even the low-lying shallow states for a tiny content of water molecules, would quench light emission or reduce the absorption. Any residual water molecules left above the surface can also cause band bending of the perovskite materials, inducing a hysteresis of the light response and utilization.

In order to investigate the dynamic behavior of MAPbI_3_ nanoclusters in water, AIMD simulations at 300 K were conducted for the small nanocluster. The simulation settings, including cluster size, number of water molecules, and box volume, are shown in Table 1. The radial distribution functions (RDFs) were calculated to provide insight into the dissolution process. In Figure 6a, the RDFs for Pb–I are plotted for each time window with a length of 2 ps. In the first time window starting at 0 ps, there are clear maximum values around 3, 7, and 9 Å, indicating that the nanocluster remains in a periodic lattice structure. However, the peaks become broadened and even eventually disappear with time, suggesting that the periodic lattice structure is destroyed, i.e., within the first 4 ps. The RDFs for N–I and N–O were also calculated. In Figure 6b, the first peak of RDF *g*_*NI*_(*r*) is in a downward trend, indicating that an increasing number of MA cations are leaving their original positions and contributing to the dissolution of the nanocluster. Conversely, Figure 6c shows that the first peak of RDF *g*_*NO*_(*r*) increases over time, indicating that water molecules capture MA cations during the dissolution process. RDFs show significant changes in the structure of the nanocluster occur in the first 4 ps.

**Figure 6:**
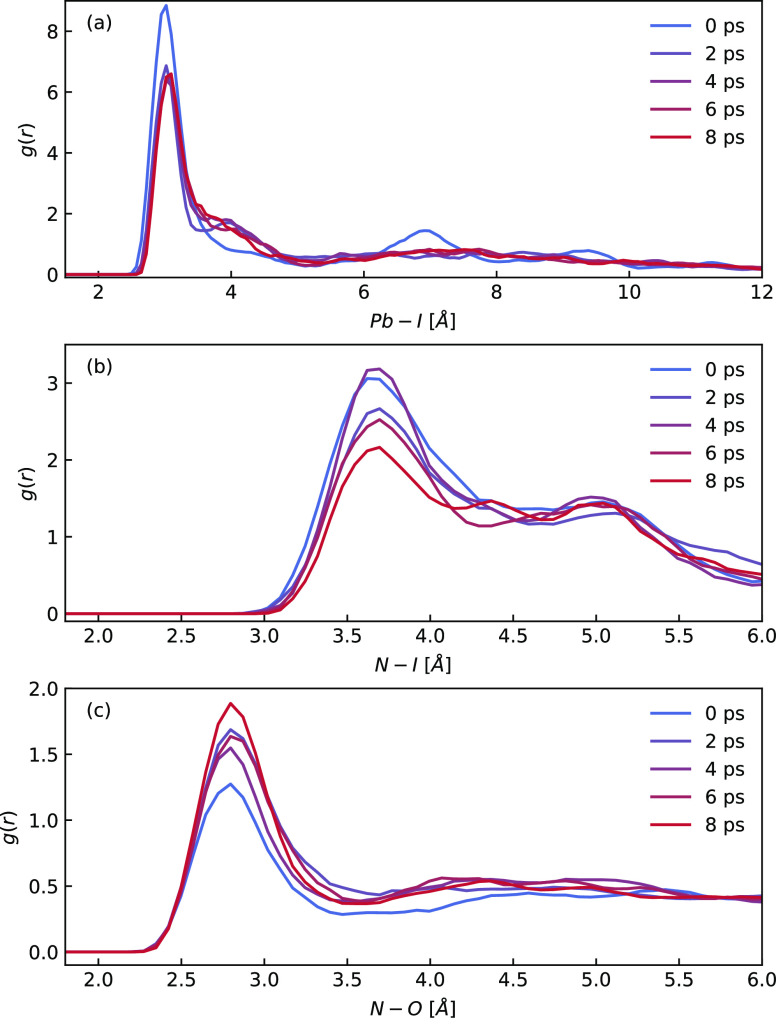
RDFs for Pb–I (a), N–I (b), and N–O (c) for each time window, with a length of 2 ps. (a) The periodic lattice structure is destroyed within the first 4 ps. (b) An increasing number of MA cations are leaving their original positions. (c) Water molecules capture the MA cations during the dissolution process.

In Figure 7, we present a series of plots that showcase the dynamic evolution of the MAPbI_3_ nanocluster configuration, topological connection of Pb–I bonds, degree distribution, and I–Pb–I angle distribution over time. The topological connection graph is generated based on the geometric criterion that the Pb–I distance is less than 3.4 Å. In the plotted graphs, the purple and gray nodes represent I and Pb atoms, respectively.

**Figure 7:**
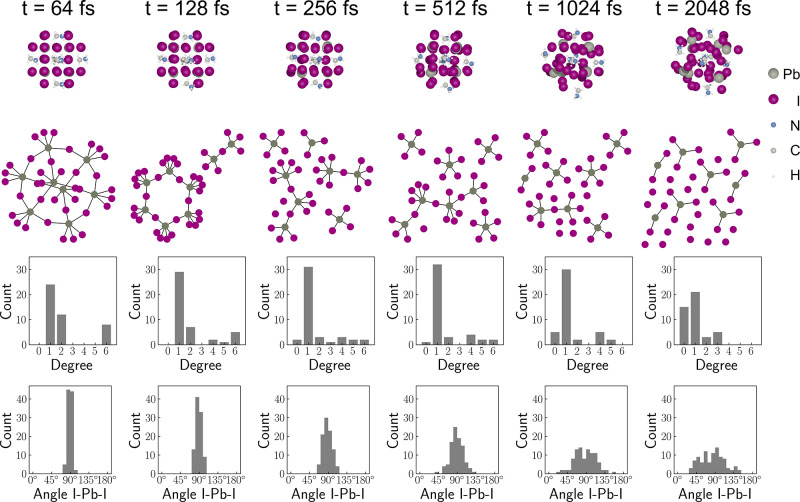
Configurations, graph structures, degree distributions, and angle I–Pb–I distributions for different frames at times 64, 128, 256, 512, 1024, and 2048 fs. The topological connection graph is generated based on the geometric criterion that the Pb–I distance is less than 3.4 Å.

Our observations indicate that the degree of Pb nodes, which signifies the number of I atoms bonded to them, decreases with time. At 64 fs, there are 8 Pb nodes with degrees of 6, indicating that each of them is bonded with 6 I atoms. However, at 1024 fs, no Pb node has a degree of 6, indicating the absence of any regular octahedron formation via the Pb and I atoms. It is worth mentioning that the newly formed Pb–I bonds are not considered in these graph plots as we only focus on the impact of water on the nanocluster’s structural integrity. Additionally, the distribution of angle I–Pb–I at 64 fs shows that most of the angles are around 90°, which is similar to the crystal structure of MAPbI_3_. However, at 2048 fs, the I–Pb–I angle distribution is vastly different from that at 64 fs. Besides, calculations of the structural similarity between the configurations at time 0 and time t in AIMD trajectories for both the small nanocluster in the water layer and bulk water (Figure S3 in SI) also indicate that the crystal structure of the small nanocluster is severely damaged within just about 2 ps. As shown in Figure S4 in the SI, we also calculated the coordination numbers for N–O, N–I, Pb–N, and Pb–I and found substantial changes in all four coordination numbers in the first 2 ps, indicating significant alterations in the nanocluster structure.

In this work, first-principles calculations were performed to reveal the size effect and the nanoscale mechanism of water molecules above the MAPbI_3_ perovskite. Working on three distinct-sized MAPbI_3_ nanoclusters, we reveal that there exists a strong band gap opening with reduced size in the perovskite nanoclusters. Through decomposed analysis, for the first time, we discover that the outermost octahedral layer of the perovskite nanocluster protects the integrity of the structure from the water layer while, surprisingly, the subsurface layers undergo more significant deviation from the bulk positions. It is likely that the degradation initiates from those subsurface layers. As subsurface layers of MAPbI_3_ nanoclusters undergo the most significant lattice expansion, it is much easier to substitute Pb or I atoms in these subsurface layers for interstitial doping. Adding any surface passivation layer containing different functional ending groups, one with enough affinity of the Pb–I in perovskite to maintain mechanical continuity, while on the other end containing a hydrophobic unit to resist the water, would help improve the stability of perovskites.

Importantly, we report the generation of water-induced defective states. Initially localized above the valence top within the band gap for the small-sized nanocluster, the defective states finally evolve into band-like states buried within the whole gap of host MAPbI_3_ for the big-sized nanocluster. Our work suggests that water molecules function as dopants and can generate hot-spot-like localized states within the gap. Even a trace of such a residual water molecule will affect light utilization and act as a trapping center for photoinduced carriers.

Furthermore, AIMD simulations show that the small-sized MAPbI_3_ nanocluster, as large as the nanocluster examined here, is highly dynamically unstable in water. The MA cations have a tendency to detach from the nanoclusters, being captured by water molecules, which finally leads to instability. Analysis of the time evolution of the breaking of the crystal structure suggests that many Pb–I bonds are broken within 2 ps. Nevertheless, clusters with bigger sizes, endowed with a higher amount of formation enthalpy, may eventually compensate for the increase of entropy and become stabilized in water.

Our work provides a new perspective on the quantum size effect and the critical role of water in the electronic structure and structural evolution of organic perovskite, which will be helpful for designing a water-resistant layer and water-included solvent for the growth of perovskite. Looking ahead, our study could inspire researchers to examine the quantum confinement effect of the perovskite nanocluster and initiate surface modification to enhance resistance to degradation by water. Furthermore, the identification of water-induced defective states within the band gap suggest compelling needs of measures of removal of these localized states, weak per molecule but massive in total, which is critical for light utilization and promoting photovoltaic efficiency of perovskite solar cells.

## Supporting Information Available

Adsorption energy calculation details; perovskite–water ratio impact on the MAPbI_3_ cluster in both structure and PDOS; and AIMD trajectory analysis including the MAPbI_3_ structure evolution and coordination number analysis (PDF)
