# Supplementary material for: Mechanism of Interaction of Water above the Methylammonium Lead Iodide Perovskite Nanocluster: Size Effect and Water-Induced Defective States
Source: J Phys Chem Lett. 2024 Jan 10;15(2):575–82. doi: 10.1021/acs.jpclett.3c02807 (PMC10809753; doi:10.1021/acs.jpclett.3c02807)
Supplement: Supplementary file 1 — jz3c02807_si_001.pdf [file jz3c02807_si_001.pdf]

Supplementary Information for Mechanism of Interaction of Water above the Methylammonium Lead Iodide Perovskite Nanocluster: Size Effect and Water-Induced Defective States

Jie Huang, Bowen Wang, Hejin Yan, and Yongqing Cai*

Joint Key Laboratory of Ministry of Education Institute of Applied Physics and Materials Engineering, University of Macau, Macau, China

E-mail: yongqingcai@um.edu.mo

1. Adsorption energy calculation details

Table S1: The energies, calculated in Atomic Units (a.u.), are used to determine adsorption energies for three systems employing the CP2K parameters EPS_SCF set to 1.0E-6 and MAX_SCF set to 150. These energies include (i). E_{total}, which is computed from the relaxed configuration of the nanocluster and water; (ii). E_{cluster}, calculated from the relaxed configuration with surrounding water molecules removed; and (iii). E_{water}, determined from the relaxed configuration with the central $MAPbI_3$ cluster removed. The adsorption energy E_{adsorb} is derived using the formula $E_{\text{adsorb}} = E_{\text{total}} - E_{\text{cluster}} - E_{\text{water}}$.

	E_{total}	E_{cluster}	E_{water}	E_{adsorb}	E_{adsorb} per H_2O
Small	-3255.99	-575.25	-2678.02	-2.71	-0.0174
Middle	-8306.63	-1948.03	-6353.72	-4.87	-0.0131
Large	-19844.96	-4533.94	-15302.18	-8.84	-0.0100

2. Perovskite-water ratio impact on the $MAPbI_3$ cluster

To explore the evolution of structure and electronic properties in $MAPbI_3$ clusters as the perovskite-water ratio increases, we focused on a small $MAPbI_3$ nanocluster as a representative example. We systematically reduced the number of surrounding water molecules, performing structure optimizations for each system. The initial structures, depicted in Figure S1 (a2)-(g2), were constructed with water molecules positioned on the lattice, as illustrated in Figure S1 (a1)-(g1), where red dots denote the sites for oxygen atoms.

The systems (a) to (g) exhibit a gradual reduction in the number of water molecules, ranging from 156 to 0. For specific details on the number of water molecules in each system, please refer to Table S2. In Figure S1, columns 3 and 4 present the relaxed structures, with and without the surrounding water molecules, respectively. The latter representation aims to facilitate a clearer observation of structural changes in the central $MAPbI_3$ clusters.

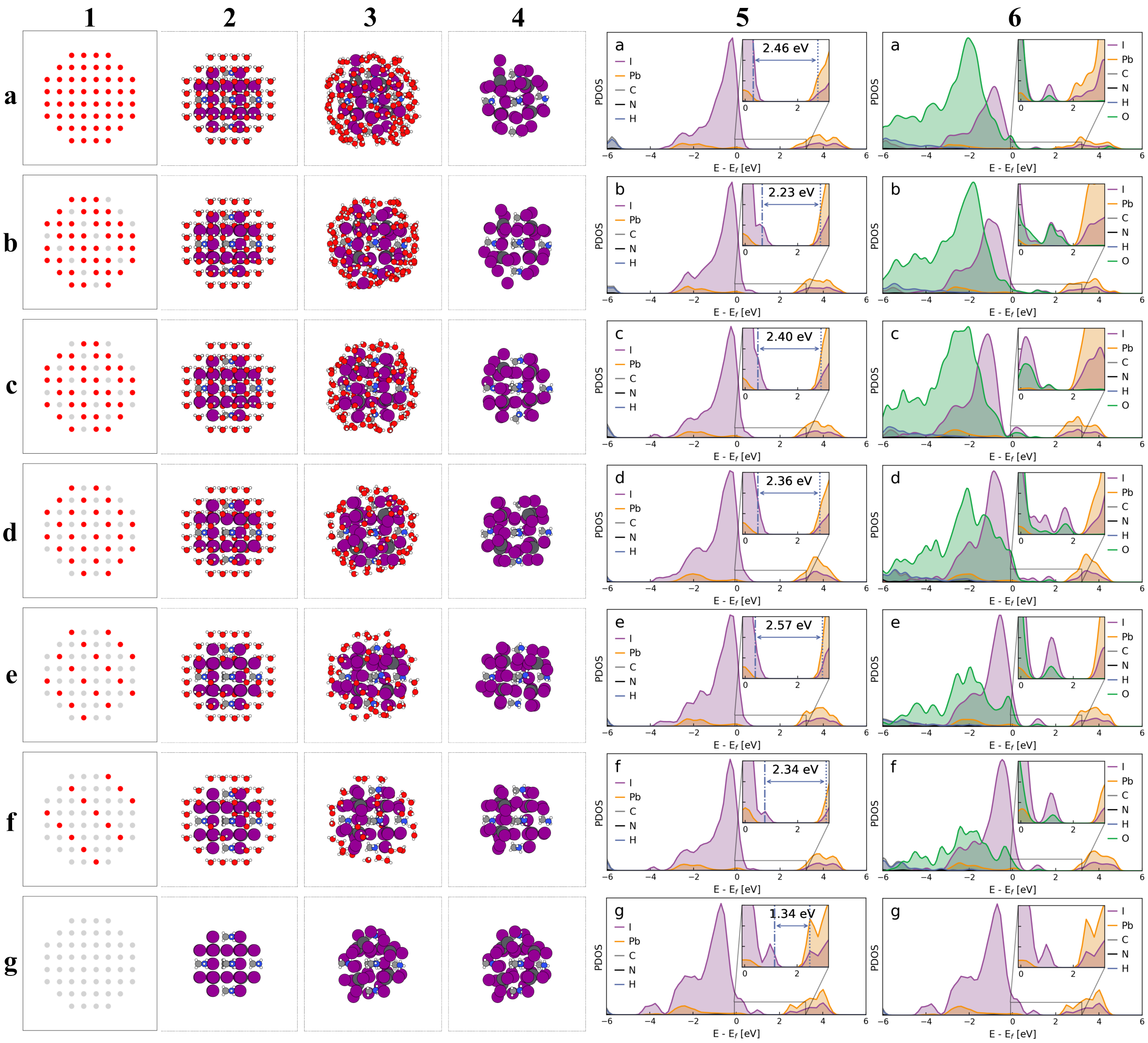

Figure S1: Evolution of $MAPbI_3$ Clusters: Investigating structural and PDOS changes with varying perovskite-water ratios. The figure illustrates the progressive reduction of water molecules, as depicted from (a2) to (g2). The surrounding water molecules are placed in ways as shown from (a1) to (g1), where red dots denote oxygen atom positions. The systems exhibit decreasing water molecules from 156 to 0. Relaxed structures with/without water molecules (columns 3 and 4) highlight changes in the central $MAPbI_3$ clusters. PDOS calculations (columns 5 and 6) investigate electronic properties. For system details, refer to Table S2.

2.1 Structural impact of water presence

In comparison to the absence of water molecules in the surrounding environment (g), the introduction of water proves beneficial in maintaining the crystalline structure of $MAPbI_3$ to some extent, even in the case of a minimal amount of water molecules, as observed in system (f). To quantitatively assess the deviation between the relaxed and initial $MAPbI_3$ structures, we calculate the cosine similarity for each cluster using the formula:

$$S(\mathbf{X}_0, \mathbf{X}_t) = \frac{\mathbf{X}_0 \cdot \mathbf{X}_t}{\|\mathbf{X}_0\| \|\mathbf{X}_t\|},$$

where $S(\mathbf{X}_0, \mathbf{X}_t)$ quantifies the similarity between two high-dimensional vectors $\mathbf{X}$ at time 0 and time t, encompassing all atom coordinates within the nanocluster.

As shown in Table S2, system (g), with no water molecules surrounding the central $MAPbI_3$ cluster, exhibits the lowest similarity, indicating the most significant structural change compared to other systems with surrounding water molecules. Intriguingly, system (f), with a minimal amount of water molecules, demonstrates the highest similarity, suggesting that system (f) preserved the best crystal structure. In other words, the influence of water molecules on stabilizing $MAPbI_3$ does not exhibit a gradual characteristic as their quantity increases.

Table S2: The number of water molecules, cosine similarities between the initial and relaxed structures, and band gaps of the naked $MAPbI_3$ when structure optimized.

System	a	b	c	d	e	f	g
Number of Water	156	117	104	78	52	39	0
Similarity $S(\mathbf{X}_0, \mathbf{X}_t)$	0.99936	0.99943	0.99947	0.99926	0.99920	0.99956	0.99904
Band gap [eV]	2.459	2.227	2.401	2.361	2.565	2.340	1.345

Furthermore, Figure S2 (a) and (b) depict structures at different optimization stages for systems (g) (no water molecules) and (f) (39 water molecules), respectively. In (a), the $MAPbI_3$ cluster in system (g) loses its octahedral structure gradually, while in (b), the basic crystal structure of the $MAPbI_3$ cluster in system (f) with a small amount of water

molecules remains preserved. This observation aligns with findings from previous works,[1–3] highlighting the stabilizing role of a small quantity of water molecules in the $MAPbI_3$ crystal structure.

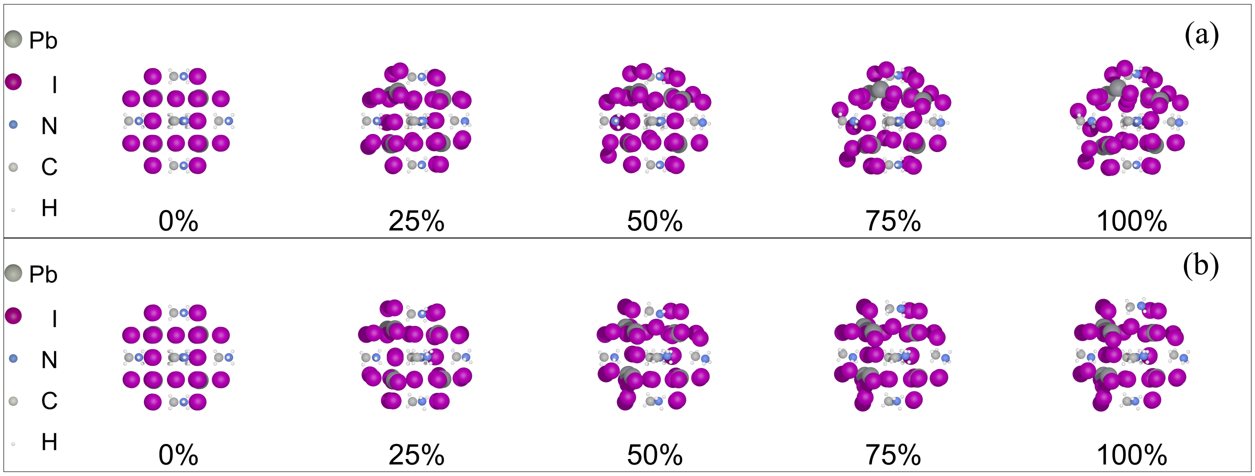

Figure S2: Structural optimization processes for (a) the naked cluster system (g) without surrounding water molecules and (b) the system (f) with 39 water molecules. In (b), water molecules are not plotted for clearer comparison. Due to differing optimization steps (240 for (a) and 361 for (b)), configurations at equivalent percentages of optimization processes are depicted. In (a), the naked nanocluster gradually loses its octahedral structure during relaxation. Conversely, in (b) with a small number of surrounding water molecules, the octahedral structure is maintained. Here (b), the water molecules play a crucial role in stabilizing the Pb-I lattice.

2.2 Electronic property impact of water presence

To investigate the impact of the perovskite-water ratio on the electronic properties of these systems, we conducted projected density of states (PDOS) calculations for both the bare clusters (Figure S1, column 4) and the cluster-water systems (Figure S1, column 3), presented in column 5 and 6 of Figure S1, respectively. Band gaps for the bare $MAPbI_3$ clusters in all systems are plotted and detailed in Table S2.

The band gaps for naked nanoclusters range from 2.23 eV to 2.57 eV, excluding system (g) without surrounding water molecules, where defective states, primarily comprised of iodine, form within the band gap of $MAPbI_3$. Examining the PDOS calculations in Figure

S1 (a6-f6) with water molecules, defective states are observed in all systems, with oxygen and iodine atoms contributing the most.

3. AIMD trajectory analysis

3.1 The evolution of $MAPbI_3$ structure

In Figure S3, we analyzed the progression of structural similarity over time in the nanocluster. We also included the nanocluster in bulk water for comparison. The results showed significant changes in structural similarity within the first 2 ps, followed by a relatively slower rate of change.

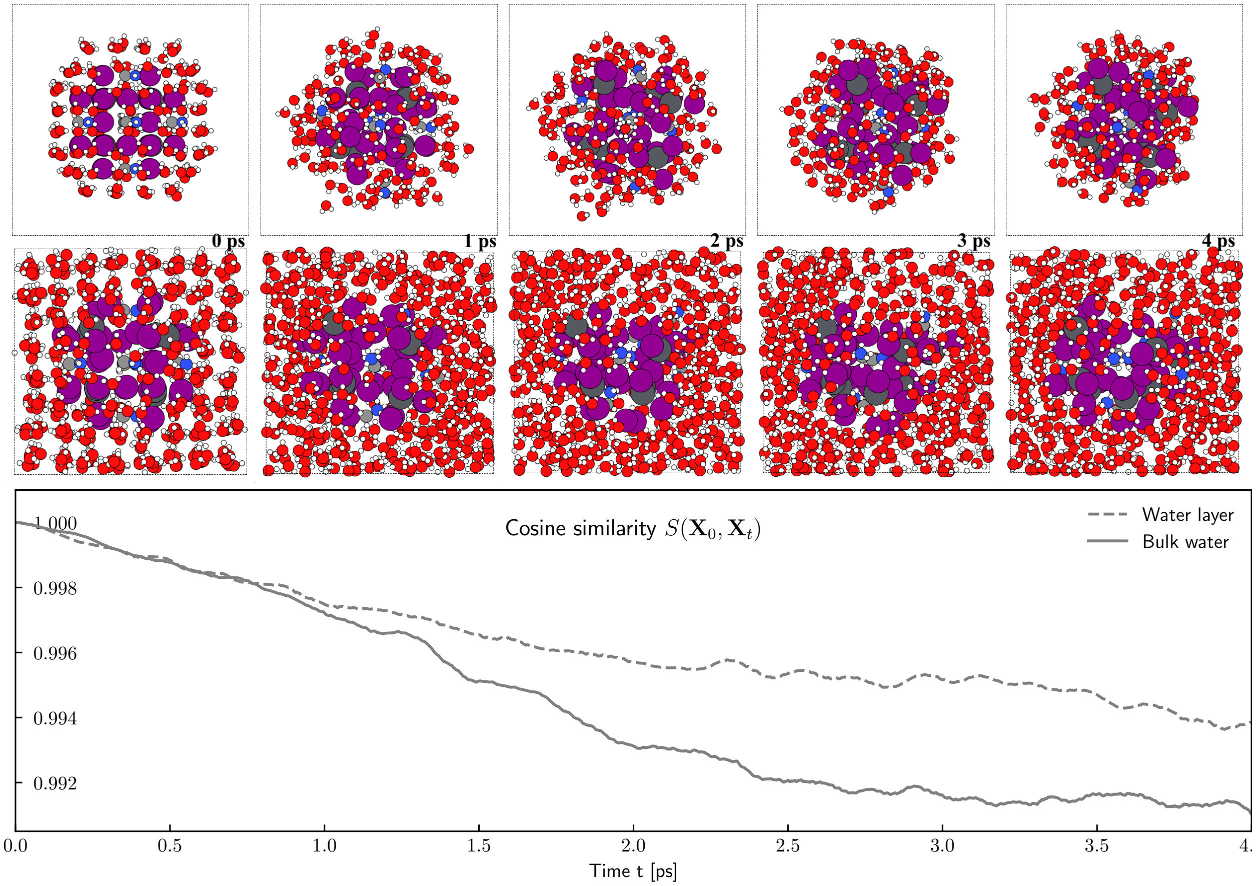

Figure S3: Snapshots of the small nanocluster configurations at the water layer and bulk water environments within the first 4 ps, as obtained from AIMD trajectories. Additionally, the corresponding cosine similarity as a function of time is also plotted.

3.2 The evolution of coordination numbers

The RDF $g_{\alpha\beta}(r)$ measures the averaged particle β density as a function of distance from a central atom α and is calculated by

$$g_{\alpha\beta}(r) = \frac{n(r)}{4\pi r^2 \cdot \Delta r \cdot \rho}$$

where $n(r)$ is the atom β number between r and $r + \Delta r$, ρ is the number density of atom β. The coordination number $CN_{\alpha\beta}$ is used to describe the number of neighbors β surrounding a reference atom α within a system and is given by

$$CN_{\alpha\beta} = \int_0^{r'} g_{\alpha\beta}(r) 4\pi r^2 \cdot \rho dr$$

where r' denotes the cutoff distance, representing the radial extent within which neighboring entities are considered. Here, the first minimum of the RDF is chosen as r'.

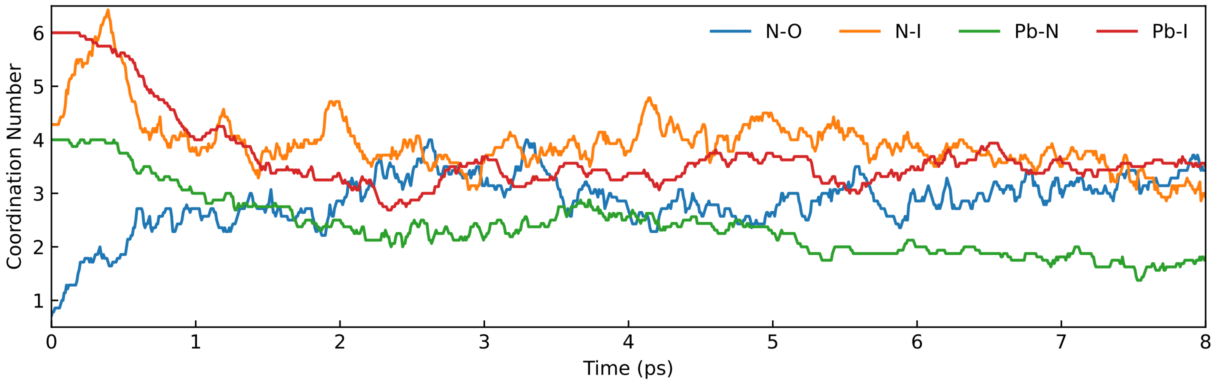

Figure S4: Time evolution of the coordination number of N-O, N-I, Pb-N, and Pb-I.

Figure S4 illustrates the time evolution of the coordination numbers for N-O, N-I, Pb-N, and Pb-I during the initial 8 ps, with cutoff values of 3.5 Å, 4.7 Å, 7 Å, and 3.7 Å, respectively. Notably, within the first 2 ps, substantial changes occurred in all four coordination numbers, indicating significant alterations in the cluster's configuration during this period.

Specifically, the coordination number N-O increased from 0.5 to 2.5 within the initial 2 ps,

suggesting a tendency for hydrogen atoms in MA cations to associate with water molecules. Conversely, the coordination numbers N-I and Pb-N exhibited a decreasing trend during this timeframe, indicating the diffusion of the MA cation away from the central $MAPbI_3$ cluster. Concurrently, the coordination number of Pb-I decreased from approximately 6 to 3, signifying a departure from the octahedral structure in $MAPbI_3$.

In contrast to prior studies[4,5] indicating the transformation of $MAPbI_3$ into monohydrate $MAPbI_3{\cdot}H_2O$ through reactions with water molecules, our observations revealed that N atoms in MA close to O atoms in water molecules during the initial picosecond, while the crystal structure of $MAPbI_3$ remained preserved. Intriguingly, this event coincided almost simultaneously with the diffusion of MA cations away from the $MAPbI_3$ cluster and the rapid loss of its crystal structure. These processes occurred nearly simultaneously without a clear time distinction.

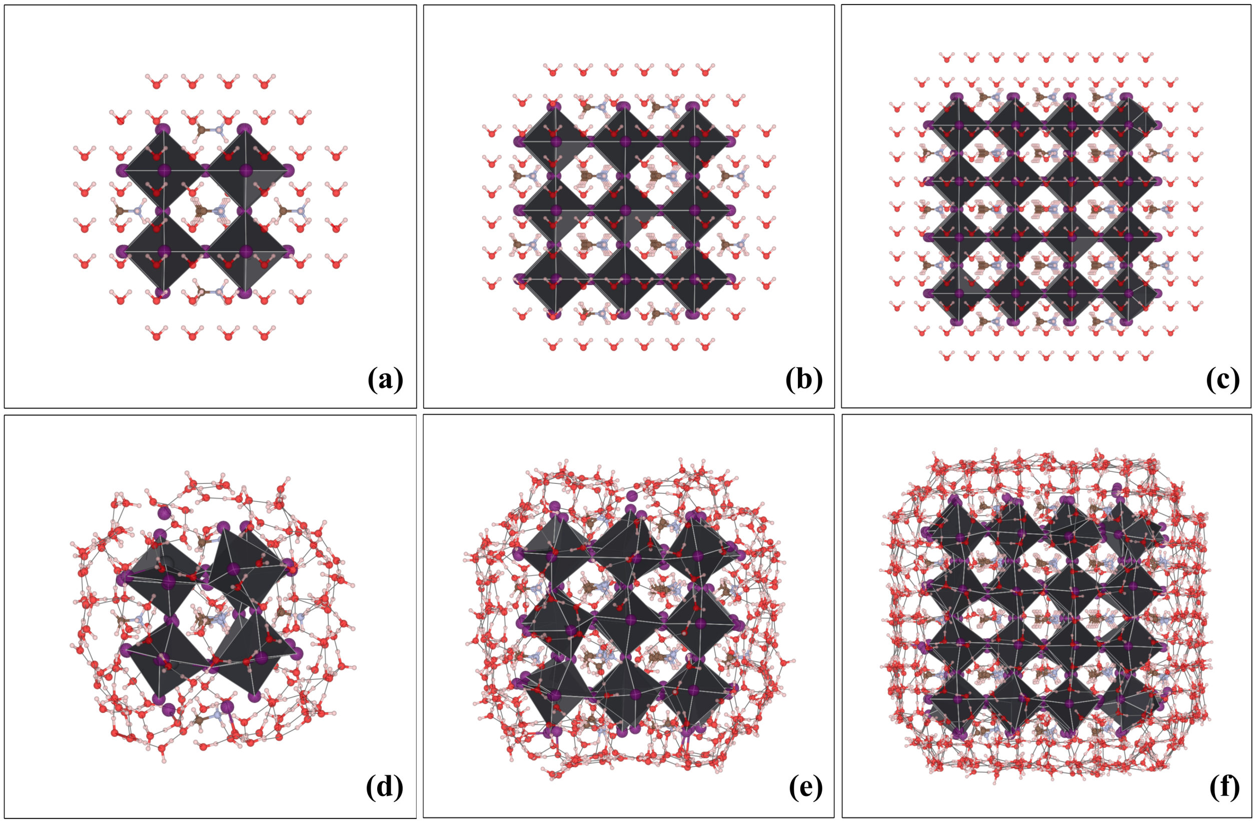

Figure S5: Comparison of the initial and relaxed structures of MAPbI3 clusters. The hydrogen networks and cluster shapes are clearly visualized. The optimized structure shows a slight increase in cluster volume and the incorporation of water molecules, forming a hydrogen bonding network around the clusters.

References

(1) Bass, K. K.; McAnally, R. E.; Zhou, S.; Djurovich, P. I.; Thompson, M. E.; Melot, B. C. Influence of moisture on the preparation, crystal structure, and photophysical properties of organohalide perovskites. *Chem. Commun.* **2014**, *50*, 15819–15822.

(2) Radicchi, E.; Ambrosio, F.; Mosconi, E.; Alasmari, A. A.; Alasmary, F. A. S.; Angelis, F. D. Combined computational and experimental investigation on the nature of hydrated iodoplumbate complexes: insights into the dual role of water in perovskite precursor solutions. *J. Phys. Chem. B* **2020**, *124*, 11481–11490.

(3) Liu, K.; Luo, Y.; Jin, Y.; Liu, T.; Liang, Y.; Yang, L.; Song, P.; Liu, Z.; Tian, C.; Xie, L. et al. Moisture-triggered fast crystallization enables efficient and stable perovskite solar cells. *Nat. Commun.* **2022**, *13*, 4891.

(4) Zhang, L.; Sit, P. H.-L. Ab initio static and dynamic study of CH3NH3PbI3 degradation in the presence of water, hydroxyl radicals, and hydroxide ions. *RSC Adv.* **2016**, *6*, 76938–76947.

(5) Zhou, X.; Jankowska, J.; Dong, H.; Prezhdo, O. V. Recent theoretical progress in the development of perovskite photovoltaic materials. *J. Energy Chem.* **2018**, *27*, 637–649.
